# Resting Electroencephalography Microstates and Alpha Power Modulation in Preschool-Aged Children with Autism Spectrum Disorder

**DOI:** 10.3390/brainsci15060544

**Published:** 2025-05-22

**Authors:** Mingxuan Ma, Ziying Yang, Leiyan Wang, Shan Lu, Junxia Han, Xiaoli Li

**Affiliations:** 1 Beijing Key Laboratory of Learning and Cognition, School of Psychology, Capital Normal University, Beijing 100048, China; 2223502033@cnu.edu.cn (M.M.); 2233502029@cnu.edu.cn (Z.Y.); 2213502103@cnu.edu.cn (L.W.); 5521@cnu.edu.cn (S.L.); 2 State Key Laboratory of Cognitive Neuroscience and Learning & IDG/McGovern Institute for Brain Research, Beijing Normal University, Beijing 100875, China; xiaoli@bnu.edu.cn

**Keywords:** autism spectrum disorder, alpha activity, EEG microstates, preschool-aged children

## Abstract

**Background/Objectives**: Emerging evidence suggests that individuals with autism spectrum disorder (ASD) exhibit altered neural connectivity and disrupted brain network dynamics, which can be captured through EEG microstate analysis. Most research to date has focused on older children, adolescents, or adults with ASD, while studies focusing on preschool-aged children with ASD remain limited. Given that early brain development is critical for understanding the onset and progression of ASD, more research targeting this age group is essential. **Methods**: In this study, resting EEG data were collected from 59 preschool-aged children with ASD and 59 typically developing (TD) participants. **Results**: The results revealed a reduction in global explained variance and coverage of microstate in children with ASD, indicating poorer social performance that was independent of alpha power after the removal of the 1/f-like aperiodic signal. These findings reflect the social symptoms commonly observed in ASD. Additionally, alpha power was found to modulate the occurrence and duration of microstates in both groups. **Conclusions**: Our findings highlight that atypical microstates can serve as reliable biomarkers for ASD, offering valuable insights into the neurophysiological mechanisms underlying the disorder and paving the way for future research directions.

## 1. Introduction

Autism spectrum disorder (ASD) is a pervasive developmental disorder characterized by repetitive, stereotyped behaviors and impairments in social interaction [1], which can significantly hinder individuals’ ability to adapt to everyday life. Abnormalities in brain activity have been linked to some of the core symptoms of ASD [2,3,4].

Alterations in specific brain regions and disruptions in the functional connectivity of brain networks can adversely affect underlying psychological processes. For instance, individuals with ASD demonstrate significantly reduced activation in the mirror neuron system and the cerebellum compared to neurotypical controls. These regions are integral to the theory of mind network, which is essential for understanding others’ thoughts and emotions [5,6]. Furthermore, the extent of disconnection within the salience network has been shown to correlate with the severity of core ASD symptoms [7]. Aberrations in the default-mode network (DMN) and its dynamic interactions with other brain networks further contribute to difficulties in integrating self-referential and social information, impairing the ability of individuals with ASD to process and relate information about themselves and others [8].

In addition to task-evoked cortical responses, intrinsic brain activity offers a complementary approach to uncovering the functional mechanisms underlying autism spectrum disorder (ASD). For instance, Itahashi et al. demonstrated that alterations in spontaneous connectivity within the posterior middle temporal gyrus (MTG) are associated with social communication impairments [9]. Furthermore, disrupted task-free activity in the right temporoparietal junction (rTPJ) has been identified as a key factor contributing to the poor mentalizing abilities observed in individuals with ASD [10]. While fMRI studies provide detailed insights into the spatial patterns of brain activity, they are limited in capturing the temporal dynamics of brain function. Given that cognition can be conceptualized as a continuous and dynamic process—akin to an uninterrupted stream—understanding the switching of brain network states is critical for a comprehensive explanation of cognitive mechanisms. In this regard, electroencephalography (EEG) microstates represent a powerful tool for examining both the temporal and spatial dimensions of brain function [11,12].

Microstates refer to quasi-stable scalp topographies that persist for brief periods, typically ranging from 80 to 120 milliseconds [13,14,15]. During brain activity, microstates rapidly alternate throughout the recording period, reflecting dynamic brain processes. These scalp maps are derived by clustering topographies sampled at peaks of global field power (GFP), and are commonly categorized into several microstate types [16]. Thus far, studies of microstates have identified several types that are tightly linked to diverse function networks. To date, microstate studies have identified distinct types associated with specific functional networks [17]. Microstate A, characterized by a right-frontal to left-posterior topography, is associated with phonological processing [18]. Microstate B, with a left-frontal to right-posterior pattern, is linked to visual processing networks [19]. Microstate C, with midline frontal–occipital distribution, is involved in self-representation and social processing [20]. Microstate D, centered in the fronto-central region, corresponds to the attention network [13].

These microstates can collectively explain up to 80% of the variance in scalp potential activity and demonstrate remarkable consistency across individuals and across the lifespan [21]. By back-fitting these microstate types to the original EEG signals, we can extract temporal information about brain network states at specific time points and the dynamic transitions between networks. The occurrence, duration, and coverage of microstates serve as key temporal parameters, while transition probability quantifies the dynamic switching properties of large-scale brain networks. Notably, aberrant microstate patterns have been linked to the neural pathophysiology of disorders such as schizophrenia, attention-deficit/hyperactivity disorder (ADHD), and depression.

Previous research has documented alterations in microstates among individuals with autism spectrum disorder (ASD). Changes in several temporal parameters, including an increased or decreased prevalence of specific microstate attributes compared to typically developing (TD) controls, suggest the presence of atypical functional brain networks [22,23]. Despite some heterogeneity in the findings, disruptions are most consistently observed in microstates B and C [24]. For example, Takarae et al. identified a significant correlation between microstate C and the severity of autistic symptoms [25]. Similarly, Bochet et al. found a significant association between microstate B and the severity of autistic symptoms [22]. Taken together, these findings suggest that microstates hold potential as candidate biomarkers for autism spectrum disorder.

Previous studies have demonstrated a strong association between alpha activity and microstates. Microstates are typically derived by extracting microstate maps from the peaks of global field power (GFP), which coincide with the alpha power peak [26]. Furthermore, the co-occurrence of microstates with alpha desynchronization suggests that alpha activity and microstates may share a common neural substrate [27]. Additionally, the frequency of microstate switching has been shown to align with twice the alpha frequency [28]. Reduced alpha activity is a well-established characteristic in children with autism spectrum disorder (ASD), and has been linked to alterations in microstate parameters [29,30]. Bochet et al. identified distinct microstate temporal parameters in toddlers with ASD during resting-state alpha band activity [22]. However, the potential moderating effect of alpha activity on the relationship between broadband microstates and autistic traits warrants further investigation. Exploring this question is crucial, as it could help to disentangle the neural underpinnings of ASD by clarifying the contributions of aberrant alpha activity and disrupted dynamic brain networks. Such insights could provide essential evidence for understanding the pathological mechanisms of ASD, ultimately supporting the development of effective neuromodulation strategies.

In addition, periodic oscillatory activity is inherently intertwined with aperiodic oscillatory activity [31,32,33]. Similarly to periodic activity, aperiodic activity also covaries with age, potentially confounding age-related findings associated with alpha rhythm activity [34,35]. In a novel contribution to the field, Hill et al. demonstrated distinct associations between microstates and alpha power in typically developing (TD) children after removing the aperiodic signal [36]. These findings underscore the importance of further investigating aperiodic-adjusted neural oscillations to better understand neurodevelopmental patterns. To address this, we parameterized alpha activity to more precisely capture individual alpha power and alpha center frequency during childhood.

In summary, this study aimed to investigate three key questions. First (Q1), we sought to determine whether distinct microstates in broadband EEG activity are present in children with ASD during early childhood. We hypothesized that significant group differences would emerge. Building on these findings, we examined (Q2) whether disrupted microstates are associated with autistic symptoms and parameterized alpha activity. Lastly (Q3), we explored whether the relationship between microstates and autistic symptoms remains robust after accounting for the influence of aperiodic-adjusted alpha activity on microstates.

## 2. Materials and Methods

### 2.1. Participants

The present study included 59 TD preschool-aged children and 59 children with ASD, aged 3–6 years and right-handed. All children with ASD were recruited and received diagnostic confirmation, based on the Diagnostic and Statistical Manual of Mental Disorders, Fifth Edition (DSM-V), by clinicians. Due to limited access to reliable information at the school site, symptom data for children with ASD were collected from parent-reported measures. Specifically, we utilized the Autism Behavior Checklist (ABC; *M* ± *SD*: 51.13 ± 16.39) and the Social Responsiveness Scale (SRS; *M* ± *SD*: 100.61 ± 22.26) to assess several symptom dimensions. Sample demographics are presented in Table 1. The study was approved by the Research Ethics Committee of the School of Psychology at Beijing Normal University and the Psychological Ethics Committee of Capital Normal University.

### 2.2. Procedure

All participating children were comfortably seated in an armchair, typically accompanied by their caregivers, in a bright and quiet room. Before the recording session, parents assisted the experimenter in helping the children to relax. During the session, children were instructed to stay alert and maintain an eyes-open state for at least 5 min. Once recording commenced, unnecessary parent–child interactions were not permitted to minimize potential distractions.

### 2.3. EEG Data Acquisition and Preprocessing

In this study, continuous open-eye resting-state EEG signals were recorded using a high-density array of 128 Ag/AgCl passive electrodes at a sampling rate of 1000 Hz. Before the EEG recording, scalp impedance was checked online by employing Net Station software version 4.5 (EGI, Inc., Eugene, OR, USA) and reduced to below 50 KΩ. EEG data were initially referenced online to Cz.

To ensure optimal spatial coverage of the frontal, central, temporal, and occipital regions, 62 electrodes of interest were selected from the 128-channel Geodesic Sensor Net (GSN). Preprocessing began with notch filtering to remove line noise, followed by band-pass filtering between 1 and 45 Hz. EEG signals were divided into non-overlapping 4 s intervals. Artifact-free segments were identified using the artifact detection algorithm proposed by Durka et al. [37], which excludes segments containing eye blinks, eye movements, muscle activity, power line interference, abrupt slopes, breathing artifacts, or outlier values. Visual inspection was then performed to further reject noisy segments. Channels exceeding a ±200 μV threshold were marked as bad and interpolated using data from neighboring channels, as described in our previous work. On average, 4.3 bad channels per recording were processed. Offline referencing was set to the average reference. After preprocessing, the average duration of EEG signals was 85.24 s in the TD group and 70.27 s in the ASD group. There was no difference in the duration of EEG signals between the ASD group and the TD group; *t* (116) = 0.17, *p* = 0.87.

### 2.4. EEG Microstate Analysis

The generation of microstates was performed using the Microstates EEGLAB toolbox 1.0 script [16]. First, 1000 global field power (GFP) peaks were identified from each individual’s EEG signal. The GFP was calculated as the standard deviation of voltage values across all electrodes at each time point, with peaks reflecting the local maxima of GFP and representing the highest signal-to-noise ratio. To ensure reliable peak selection, a minimum interval of 10 ms between peaks was set, and peaks below a value of 20 µV or those falling outside ±1 standard deviation were excluded. Brain maps corresponding to GFP peaks were then clustered using a modified K-means algorithm (ignoring polarity) to identify microstates across a range of 2–8 clusters. For each clustering solution, 50 random initializations were performed, with a maximum of 2000 iterations. The number of microstate clusters was determined based on global explained variance (GEV) and cross-validation (CV) criteria. GEV quantified the similarity between EEG samples and their assigned microstate prototypes, while CV assessed residual noise. The optimal number of clusters was selected by maximizing the GEV and minimizing CV, achieving a balanced trade-off between cluster quantity and model fit. A solution with five clusters was chosen to achieve this balance while maintaining generalizability (4 clusters: normalized CV = 0, normalized GEV = 0.54; 5 clusters: normalized CV = 0.03, normalized GEV = 0.7; 6 clusters: normalized CV = 0.24, normalized GEV = 0.81).

Following clustering, microstates were back-fitted to the original EEG signals, producing a sequence of five labeled microstate types: MS A, MS B, MS C, MS D, and MS E. Each label represented the dominant microstate at a given time point. Collectively, the identified microstates accounted for approximately 61–71% of the variance in EEG data.

Based on the labeled microstate sequences, the following microstate parameters and transition probabilities were computed:

Coverage: the proportion of time each microstate was active for in the EEG signals.

Occurrence: the average number of times per second that a microstate dominated. 

Duration: the average length of time a given microstate persisted for.

GFP: the local maxima of global field power associated with each microstate.

Transition probability (TP): the likelihood of transitioning from one microstate to another, modeled as a first-order Markov chain process.

These parameters provide insights into the temporal dynamics and functional organization of brain activity as captured through EEG microstates.

### 2.5. Characterization of Alpha Activities

The parameterization of the spectral data was divided into two steps. First, we used Welch’s method (Hamming windowing with 50% overlap), implemented in Matlab 2019b, to calculate the power spectral density (PSD) for each participant by averaging all electrodes. Furthermore, each subject’s PSD was packaged using the fitting oscillations and the one-over-f (FOOOF) Python toolbox version 1.0.0: https://fooof-tools.github.io/fooof/ (accessed on 15 January 2024) to compute the parameterization of alpha activities by separating periodic and aperiodic components of PSD data [32]. In the FOOOF algorithm, periodic power was defined as a “bump” above the aperiodic signal, while the center frequency corresponded to the frequency under the peak of the bump. For this study, we reconstructed PSD signals in the broad frequency range between 1 and 40 Hz without using a knee, resulting in a goodness-of-fit exceeding 0.95 and a residual error below 0.03. Next, we detected the periodic power in the entire signal. The settings for the algorithm were as follows: peak width limits = [1, 10], maximum number of peaks = 10, peak threshold = 1, and minimum peak height = 0. Finally, we limited the alpha band to 8–12 Hz to extract the parameterized alpha power and center frequency. There were 94.07% alpha peaks of subjects falling within this range.

### 2.6. Statistical Analysis

Outliers in the dataset, defined as values falling below the lower quartile or above the upper quartile, were identified as extreme values. Data exclusion was based on a stringent threshold: if a participant’s data contained extreme values in more than three instances across all four temporal parameters (coverage, duration, occurrence, and GEV) for the five microstate types (MS types), their data were excluded. Similarly, for transition probabilities (TP types), data were excluded if extreme values occurred more than four times across all TP types. Here, we identified 3 outliers in MS types and 2 outliers in TP types.

To address Q1, three-way mixed analysis of variance (ANOVA) with Type III tests, supported by the BruceR package in R version 2024.04.0, was employed to compare group differences. The independent variables included subject type (ASD vs. TD), MS type, and gender, while the dependent variables consisted of the four temporal parameters (coverage, duration, occurrence, and GEV) and transition probabilities. Additionally, a two-way between-subjects ANOVA was performed to examine group differences, with subject type and gender as independent variables. The Greenhouse–Geisser correction was applied when the sphericity assumption was violated. We applied the Scheffé correction to the post hoc tests to control the Type I error rate, given that we did not decide which pairs would be compared in advance. Although the data were almost non-normal (skewness *(M* ± *SD*): 0.66 ± 0.45, kurtosis (*M* ± *SD*): 1.23 ± 0.84), our sample size was sufficiently large to ensure the stability of the results. Importantly, the overall findings remained consistent after applying data transformations. For simplicity, the results are presented based on untransformed data. The Box–Cox method was utilized to identify optimal parameters for normal transformation, but was not applied in the reported analyses for clarity.

To investigate Q2, a linear mixed model (LMM), supported by the lmer Test package in R, was used to examine the relationship between microstates and alpha activity. Subject type (ASD vs. TD) and alpha activity were included as fixed factors, while MS type was incorporated as a random intercept. The model satisfied assumptions of multicollinearity.

Finally, Pearson’s correlation analysis was conducted to evaluate the association between microstate parameters and clinical scale scores. We checked all variables and found that the total score of ABC and the total score of SRS violated statistical assumptions. Therefore, we used Spearman’s rank correlation to examine the relationship between these two variables and other microstate parameters. The False Discovery Rate (FDR, n = 6) correction was applied to account for multiple testing. For Q3, semi-partial correlations were computed to assess the relationship between microstates and clinical outcomes, while controlling for the moderating effects of alpha activity on MS parameters. The statistical significance was assessed using a two-tailed approach across for all analyses.

## 3. Results

### 3.1. Abnormal Aperiodic-Adjusted Alpha Activity and Microstate Parameters in ASD

#### 3.1.1. Aperiodic-Adjusted Alpha Activity

We conducted a two-way ANOVA to examine group differences in alpha power and alpha center frequency, controlling for gender differences. The results indicated that children with ASD exhibited lower alpha power and alpha center frequency. However, the difference in alpha power did not reach statistical significance (alpha power: *F* (1105) = 1.24, *p* = 0.27, η^2^_p_ = 0.01; alpha center frequency: *F* (1105) = 4.02, *p* < 0.05, η^2^_p_ = 0.04).

#### 3.1.2. Microstate (MS) Analysis 

**Figure 1:**
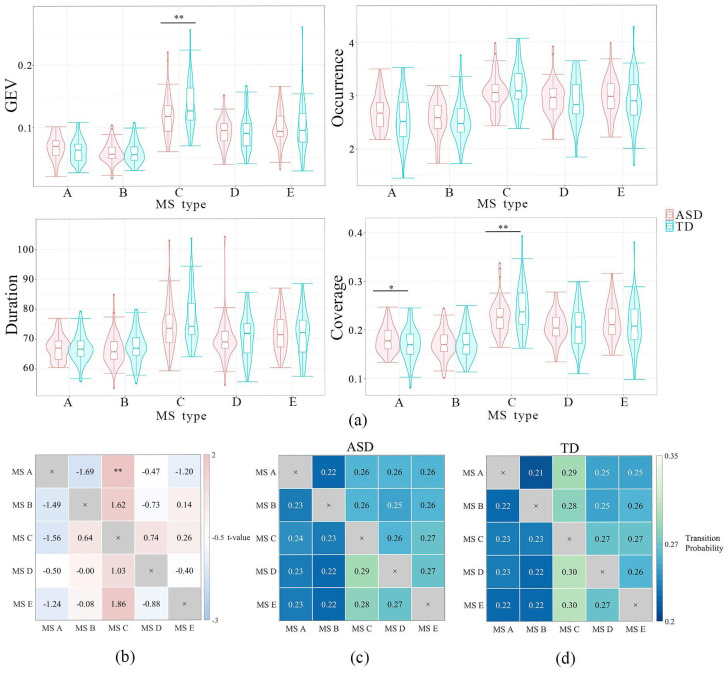
Between-group differences in the five MS parameters, including transition probability. (**a**) Between-group differences in the five MS parameters (TD–ASD); (**b**) t-values of between-group differences in transition probability; (**c**) the transition probability of the ASD group; and (**d**) the transition probability of the TD group. * *p* < 0.05, ** *p* < 0.01.

A three-factor ANOVA was performed to examine the main effect of subject type (ASD vs. TD) on microstate (MS) parameters. Given that the three-way interaction was not significant, we compared MS parameters between the ASD group (n = 57) and the TD group (n = 56) with the Scheffé correction. The most notable differences were observed in MS C and MS A (see Figure 1). Specifically, children with ASD demonstrated lower global explained variance (GEV) in MS C (*t* (109) = 3.03, *p* = 0.003, Cohen’s d = 0.67), lower coverage in MS C (*t* (109) = 2.42, *p* < 0.02, Cohen’s d = 0.45), and higher coverage in MS A (*t* (109) = 2.89, *p* = 0.03, Cohen’s d = 0.53).

For transition probabilities (TPs), significant group differences were found between MS A and MS C. Children with ASD (n = 57) had a lower transition probability between these MS types compared to the TD group (n = 57) (*t* (112) = –3.03, *p* = 0.003, Cohen’s d = 0.48).

In all the above analyses, no significant interaction effects were observed between subject type and gender. While other significant results were identified in individual MS types, these findings are not detailed here to maintain focus on the study’s primary aims.

### 3.2. Aberrant Temporal Dynamics of Microstates Related to ASD Symptoms and Social Performance

**Figure 2:**
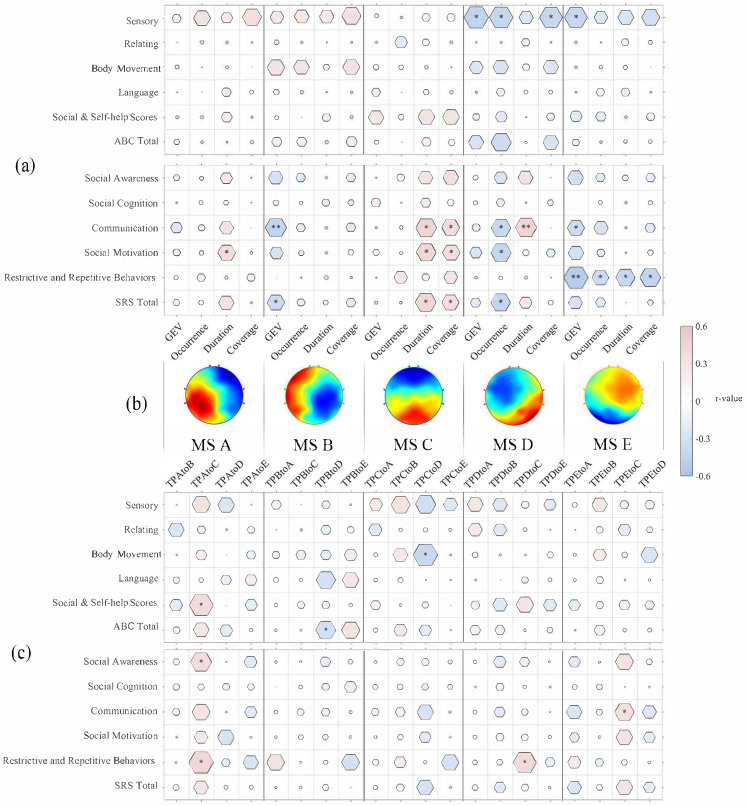
Correlations between the five MS parameters and autistic characteristics. (**a**) The r-values between MS parameters and ABC and SRS scores; (**b**) brain maps of the five microstates; (**c**) the r-values between MS transition probabilities and ABC scores and SRS scores. * *p* < 0.05, ** *p* < 0.01. Abbreviations: ABC: Autism Behavior Checklist; SRS: Social Responsiveness Scale.

Correlations between MS parameters and clinical measures were assessed using Pearson’s correlation (see Figure 2).

#### 3.2.1. Microstate A

A significant positive correlation was found between coverage and SRS social motivation (*r* = 0.36, *p* = 0.03).

#### 3.2.2. Microstate B

A significant negative correlation was found between the GEV of MS B and SRS communication scores (*r* = −0.36, *p* = 0.03).

#### 3.2.3. Microstate C

A significant positive relationship was identified between the duration and coverage of MS C and both SRS communication and social motivation scores. Specifically, duration was positively correlated with SRS communication scores (*r* = 0.42, *p* < 0.01) and SRS social motivation scores (*r* = 0.40, *p* < 0.02), and coverage was positively correlated with SRS communication scores (*r* = 0.36, *p* < 0.03) and SRS social motivation scores (*r* = 0.35, *p* < 0.04).

#### 3.2.4. Microstate D

The duration of MS D was positively related to SRS communication scores (*r* = 0.44, *p* < 0.01), while its occurrence was negatively related to SRS communication scores, as well as SRS social motivation scores (communication scores (*r* = –0.40, *p* < 0.02); social motivation scores (*r* = –0.42, *p* = 0.01)).

For the ABC scale, the GEV, occurrence, and coverage for MS D were negatively correlated with ABC sensory scores (GEV (*r* = –0.43, *p* < 0.02); occurrence (*r* = –0.41, *p* = 0.02); coverage (*r* = –0.40, *p* < 0.03)).

#### 3.2.5. Microstate E

For MS E, all parameters were negatively related to SRS restrictive and repetitive behavior scores (GEV (*r* = –0.50, *p* = 0.002); occurrence (*r* = –0.34, *p* = 0.04); duration (*r* = –0.39, *p* < 0.02); coverage (*r* = –0.42, *p* = 0.01)). The GEV of MS E was negatively related to SRS communication scores (*r* = –0.36, *p* = 0.03).

For the ABC scale, a significant negative correlation was found between the GEV and ABC sensory scores for MS E (*r* = –0.40, *p* < 0.03).

Analysis of transition probabilities revealed that TP A to TP C was positively associated with several clinical measures (social and self-help scores (*r* = 0.38, *p* = 0.02), social awareness scores (*r* = 0.36, *p* = 0.03), and restrictive and repetitive behavior scores (*r* = 0.42, *p* = 0.01)). Furthermore, TP E to TP C demonstrated a significant positive correlation with communication scores (*r* = 0.32, *p* < 0.05).

### 3.3. Independence of the Effect of Microstates on ASD Symptoms

#### 3.3.1. Alpha Activity Modulated Microstates

We first investigated whether alpha activity modulated microstate (MS) parameters. The results showed that alpha power had a significant main effect on the occurrence (*F* (1532) = 19.35, *p* < 0.001) and duration (*F* (1532) = 39.23, *p* < 0.001) of microstates. The lack of significant interaction effects (*p*-values > 0.12) indicated that alpha power modulated the occurrence and duration consistently across both the ASD and TD groups. However, no significant main effects of alpha power were observed for coverage and GEV (*p*-values > 0.68). Similarly, alpha central frequency showed no significant effects on any MS parameters (*p*-values > 0.08).

#### 3.3.2. Semi-Partial Correlation

To assess the independence of the relationship between MS parameters and clinical measures from alpha activity, we computed semi-partial correlations, accounting for the effects of alpha power. This analysis focused on MS parameters previously linked to Autism Behavior Checklist (ABC) and Social Responsiveness Scale (SRS) scores: the duration of MS A, MS C, MS D, and MS E, and the occurrence of MS D and MS E. The results demonstrated that most correlations remained significant even after controlling for the influence of alpha power on MS parameters (see Table 2). These findings suggest a robust and stable relationship between microstate parameters and clinical symptoms, underscoring the reliability of the observed associations.

## 4. Discussion

The primary objective of this study was to identify aberrant microstates (MSs) in preschoolers with ASD, and to explore the impact of alpha activity on the relationship between microstates and autistic characteristics. Our findings revealed elevated MS A and diminished MS C in children with ASD, along with a reduced dynamic transition from MS A to MS C. Notably, several anomalous microstates were significantly associated with clinical symptoms. These associations remained robust even after controlling for the influence of alpha activity on microstate-scale correlations.

In line with previous research on adults [11,38], we identified five distinct microstates in preschoolers: MS A: associated with auditory processing, and linked to activity in the left–middle and superior temporal lobes [18]; MS B: corresponding to the visual network, with strong activation in the cuneus and primary visual cortex [19]; MS C: generated in parietal brain regions, and pivotal for processing social information and self-referencing [39]; MS D: related to the attention network and bottom-up attention, and located in the right-frontal lobe [40,41]; and MS E: associated with the sensorimotor network, with activity localized in the cerebellum [17].

Most studies have reported a lower prevalence of microstate (MS) C in individuals with autism spectrum disorder (ASD) [24,42]. The present study extends these findings from a sample of 7-year-olds to the preschool period. However, the study by [22] identified atypical microstate parameters in toddlers with ASD primarily in microstate B. Such age-dependent variations suggest a developmental trajectory of microstates with age, with the preschool period potentially serving as a critical window of change. The developmental trajectory linking microstate parameters to their association with clinical manifestations remains to be further validated in samples spanning a continuous age range.

Takarae et al. observed reduced global explained variance (GEV) of MS C in individuals with high-functioning ASD [25]. Consistent with their findings, our study also identified lower GEV for MS C in preschool-aged children with ASD. GEV refers to the percentage of total variance in the data explained by a given microstate. Coverage, on the other hand, represents the fraction of total recording time during which the microstate is dominant [43], reflecting the relative temporal engagement of its underlying neural generators. A decrease in GEV for MS C may be attributed to reduced coverage of the same microstate. In our study, children with ASD spent less time in MS C during resting states, as indicated by the observed lower coverage. Reduced activity in the default-mode network (DMN), represented by MS C, could lead to impairments in processing social information [8,44]. Furthermore, consistent with [25], we found that reduced duration of MS C serves as a significant predictor of the severity of social symptoms. However, our findings also revealed a positive relationship between MS C and Social Responsiveness Scale (SRS) scores, which initially seems counterintuitive, given the lower MS C observed in children with ASD compared to typically developing (TD) preschoolers. This discrepancy is not unprecedented. For instance, Gui et al. found lower MS 4 (analogous to MS C in our study) in individuals with ASD under no-stimulation conditions, which was positively correlated with individual social performance [45]. Therefore, these results should be considered as an indicator of hyper-responsiveness, rather than an accident or statistics error. Over-responsiveness occurs when a child responds to sensory input more quickly or with increased intensity than typically observed [46]. Atypical patterns of sensory responsiveness have been identified in 69–100% of children with ASD in various studies [47,48]. Overwhelming sensory responsiveness can not only predict social severity [49], but also be related to stereotyped behaviors [50]. Reduced MS C may reflect a compensatory mechanism for this hyperactivation.

Notably, the global explained variance (GEV), occurrence, and coverage of MS D were significantly negatively correlated with sensory scores. Atypical sensory perception, such as an increased focus on details, is well documented in individuals with ASD. These perceptual abnormalities may stem from a lack of modulation by higher-order conceptual processing or “meaning”. Our findings suggest that atypical sensory processing in ASD might be linked to disruptions in the attention network represented by microstate D [51].

Additionally, all temporal parameters of MS E were significantly negatively correlated with restrictive and repetitive behavior scores. Prior research has shown that MS E is associated with the sensorimotor network, which includes key regions such as the inferior parietal lobe and the cerebellum [17]. Children with ASD often display atypical activity in these regions. This finding suggests that disruptions in the sensorimotor network, as represented by microstate E, may underlie the development of stereotypical behaviors in children with ASD.

Consistently with previous studies, we found that alpha power modulated several microstate (MS) parameters [27,36]. These effects are consistent across groups, but not ASD-specific. Increased alpha power during spontaneous activity may reflect conscious cognitive processes responsible for microstate generation [17,52]. Ref. [27] demonstrated that alpha power is associated with the occurrence of microstates and plays a role in maintaining and facilitating the switching between different microstates. Interestingly, Takarae et al. [27] identified a relationship between alpha power and microstates only in typically developing (TD) individuals, suggesting that anomalous microstates might underlie the attenuated alpha power observed in autism spectrum disorder (ASD) [25]. In the present study, we parameterized alpha power and observed significant main effects in both groups.

Notably, research focusing on parameterized alpha power in autism remains extremely limited [53]. Considering that both periodic and aperiodic EEG activity are subject to developmental changes [34,35] and vary between typical and atypical developmental trajectories [31,54,55], it is crucial for future autism research to place greater emphasis on the parameterization of both periodic and aperiodic activity. This should include investigations into their relationship with microstates and other neural markers to enhance our understanding of neurodevelopmental mechanisms in ASD. In the current study, we did not find significant group differences in alpha power, which may be influenced by sample heterogeneity and methodological variations. Our use of parameterized alpha power, which has been less commonly adopted in prior studies, might also contribute to the discrepancy with respect to previous findings. Nonetheless, given the established link between aberrant alpha oscillations and a range of clinical manifestations, conducting further controlled analyses would still be valuable.

We initially controlled the effect of alpha power on microstates to isolate the pure relationship of microstates on autistic characteristics. Semi-partial correlations revealed stable relationships between microstates and clinical measures, underscoring their potential as biomarkers for ASD. This stability suggests that microstates could guide targeted interventions. For example, transcranial alternating current stimulation (tACS) at 10 Hz has shown promise in treating ADHD [56]. However, for ASD, interventions may need to target other frequency bands, given the unique neural dynamics associated with the disorder [57,58].

A potential limitation of the present study is the lack of detailed information regarding participants’ current medication use, comorbid conditions (e.g., ADHD), and intellectual functioning. These factors have been shown to influence EEG patterns and may, therefore, act as potential confounding variables in the interpretation of our findings. Although all participants were clinically diagnosed by experienced professionals, future studies should include more comprehensive clinical assessments and consider these variables as covariates in the analysis to better isolate the specific neural correlates of autistic traits.

## 5. Conclusions

Our study highlights the aberrant microstate dynamics in preschoolers with ASD, emphasizing the interplay between alpha activity and microstates in shaping autistic characteristics. These findings pave the way for further research into microstate-based interventions and their potential to alleviate core symptoms of ASD in early development.

## Figures and Tables

**Table 1:** Demographic information of all study participants.

Characteristic	ASD	TD
Age (*M* ± *SD*)	4.59 ± 1.00	4.64 ± 0.97
Gender (male/female)	47/12	47/12
ABC score (*M* ± *SD*)	51.13 ± 16.39	–
SRS score (*M* ± *SD*)	100.61 ± 22.26	–

**Table 2:** Comparison of correlation results and semi-partial correlation results between selected microstate parameters and clinical scores, accounting for the effects of alpha activities on microstates.

MS Type	MS Parameters	Correlation	Clinical Scores					
			Sensory	Relating	Body Movement	Language	Social and Self-Help	ABC Total Score
A	Occurrence	*r*	0.29	−0.06	0.02	−0.01	−0.06	0.26
		semi-*r*	0.32	−0.03	0.05	0.01	−0.03	0.09
	Duration	*r*	0.2	0.05	−0.06	−0.17	0.19	−0.02
		semi-*r*	0.18	0.02	−0.09	−0.19	0.17	0
C	Occurrence	*r*	0.05	−0.22	0.09	−0.01	0.11	0.07
		semi-*r*	0.04	−0.23	0.08	−0.02	0.1	−0.02
	Duration	*r*	0.15	0.13	−0.07	0.11	0.29	−0.09
		semi-*r*	0.18	0.18	−0.02	0.13	0.33	0.22
D	Occurrence	*r*	−0.41	−0.04	−0.27	−0.15	−0.2	−0.29
		semi-*r*	−0.4	0.01	−0.24	−0.13	−0.18	−0.31
	Duration	*r*	−0.25	0.15	−0.13	0.08	0.02	−0.08
		semi-*r*	−0.28	0.11	−0.17	0.06	−0.01	−0.09
E	Occurrence	*r*	−0.24	0.02	−0.06	0.15	−0.18	0.02
		semi-*r*	−0.28	−0.03	−0.12	0.13	−0.23	−0.11
	Duration	*r*	−0.28	0.14	0.08	−0.16	−0.05	−0.14
		semi-*r*	−0.34	0.07	0.01	−0.21	−0.11	−0.16
			**Social Awareness**	**Social Cognition**	**Communication**	**Social Motivation**	**Restrictive** **and Repetitive Behaviors**	**SRS Total Score**
A	Occurrence	*r*	−0.09	−0.09	−0.14	−0.1	0.17	0.04
		semi-*r*	−0.08	0.01	−0.1	−0.03	0.12	−0.05
	Duration	*r*	0.25	0.11	0.29	0.36 *	0.07	0.21
		semi-*r*	0.25	0.09	0.28	0.35 *	0.08	0.31
C	Occurrence	*r*	0.16	−0.02	0.08	0.08	0.27	0.16
		semi-*r*	0.16	−0.05	0.07	0.06	0.29	0.05
	Duration	*r*	0.29	0.21	0.43 *	0.4 *	0.16	0.40 *
		semi-*r*	0.3	0.24	0.44 *	0.43 *	0.14	0.44 *
D	Occurrence	*r*	−0.11	−0.09	−0.17	−0.25	−0.06	−0.23
		semi-*r*	−0.11	−0.1	−0.18	−0.26	−0.05	−0.21
	Duration	*r*	−0.27	−0.18	−0.4 *	−0.42 *	−0.08	−0.40 *
		semi-*r*	−0.26	−0.14	−0.38 *	−0.39 *	−0.11	−0.39 *
E	Occurrence	*r*	−0.19	0.09	−0.29	−0.17	−0.34 *	−0.19
		semi-*r*	−0.24	0	−0.38 *	−0.27	−0.32	−0.3
	Duration	*r*	−0.13	0.05	−0.05	−0.03	−0.39 *	−0.15
		semi-*r*	−0.15	−0.02	−0.09	−0.09	−0.37 *	−0.04

Note: * *p* < 0.05.

## Data Availability

The analyses presented here were not preregistered. Study materials, data, and code for replicating the study and its analyses are not publicly available, but can be accessed upon reasonable request to the corresponding author.

## Abbreviations

The following abbreviations are used in this manuscript:

ASD	Autism spectrum disorder
EEG	Electroencephalography
ABC	Autism Behavior Checklist
SRS	Social Responsiveness Scale
MS	Microstate
TP	Transition probability
GEV	Global explained variance
GFP	Global field power
